# Photophysics of tetracarboxy-zinc phthalocyanine photosensitizers

**DOI:** 10.1039/d2ra05676c

**Published:** 2022-11-07

**Authors:** Tamara Potlog, Ana Popusoi, Ion Lungu, Stefan Robu, Ion Bulimestru

**Affiliations:** Physics Department and Engineering, Moldova State University Chisinau Moldova ionlungu.usm@gmail.com tpotlog@gmail.com; The Faculty of Chemistry and Chemical Technology, Moldova State University Chisinau Moldova

## Abstract

Zinc-tetracarboxy-phthalocyanine (ZnPc(COOH)_4_) was synthesized by a melting method and basic hydrolysis. A ZnPc(COOH)_4_/Fe_3_O_4_/Ch composite was prepared by immobilization of ZnPc(COOH)_4_ onto Fe_3_O_4_/chitosan nanoparticles by a simple immersion method. The photophysical properties were studied using UV-vis spectrophotometry, fluorescence spectroscopy and time-correlated single photon counting (TCSPC) in different aqueous solutions. The UV-vis spectra of the ZnPc(COOH)_4_/Fe_3_O_4_/Ch composite displays absorption by the aromatic rings, with a Q band exhibited at *λ*_max_ = 702 nm. Moreover, the ZnPc(COOH)_4_/Fe_3_O_4_/Ch composite exhibits long triplet-state lifetimes of 1.6 μs and 12.3 μs, crucial for application as a photosensitizer. A triplet quantum yield of 0.56 for the ZnPc(COOH)_4_/Fe_3_O_4_/Ch composite in DMSO/H_2_O was achieved. FTIR showed that the conjugation of ZnPc(COOH)_4_ with Fe_3_O_4_/chitosan nanoparticles was achieved by electrostatic interaction.

## Abstract 2

A ZnPc(COOH)_4_/Fe_3_O_4_/Ch photosensitizer was synthesized by immobilization of ZnPc(COOH)_4_ onto Fe_3_O_4_/chitosan nanoparticles by a simple immersion method.

## Graphical abstract

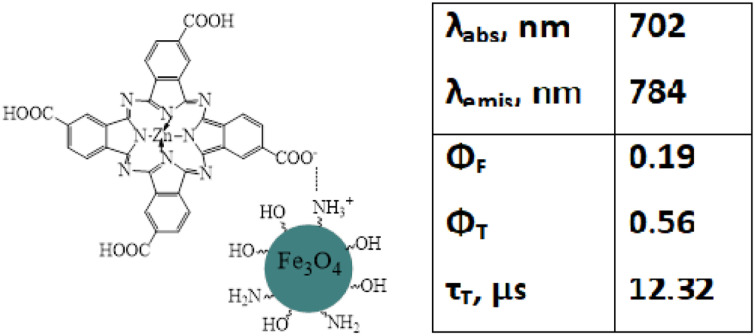

## Introduction

Metallophthalocyanine (MPc) derivatives are popular photodynamic therapy (PDT) photosensitizers (PSs). Research on a novel PS requires extensive human effort and a high cost investment over decades before clinical application. Nevertheless, some MPc derivatives, such as: aluminium phthalocyanine (Photosens®, Russia), used against skin, breast and lung malignancies, and cancers of the gastrointestinal tract;^1^ silicon Pc (Pc 4, USA), for the sterilization of blood components against human colon, breast and ovarian cancers, and gliomas;^2^ and a liposomal zinc phthalocyanine formulation, using a controlled organic solvent dilution against squamous cell carcinomas of the upper aerodigestive tract,^3^ have undergone clinical trials.

Current efforts are being made in the development of new photosensitizers (PSs) with improved solubility in body fluids and injectable solvents, photostability, enhanced permeability and retention effect, elimination and cumulative systemic toxicity.^4–9^ In the field of organic photosensitizers, metallophthalocyanines (MPcs) play an important role due to their excellent photo- and electro-chemical stability and exclusive light-harvesting capability in the red/NIR spectral regions.^10–13^

The main disadvantages of MPcs in PDT are the lack of solubility and selectivity; therefore, the combination of magnetic iron oxide nanoparticles with a photosensitizer is a new and promising approach in PDT. Fe_3_O_4_ nanoparticles have been successfully applied in tumor therapy by inducing hyperthermia and oxidative stress that lead to tumor cell damage.^14–16^ For application in PDT, magnetic nanoparticles (NPs) are usually coated with polymers, bound to the particle through organic linkers.^17^ Functionalization of Fe_3_O_4_ nanoparticles may lead to enhancement of their biocompatibility, colloidal stability, and an increase in the number of groups, through which the required antitumor effect can be obtained.

The major goal of this paper is to create a new photosensitizer with adequate solubility, especially in body fluids and injectable solvents, with greater tumor selectivity, enhanced hydrophilicity, and strong absorption in the NIR spectral region. Therefore, conjugation of an MPc derivative to a magnetic NP functionalized with a polymer is the first part of our research aimed at delivering PSs to tumor cells. The magnetic iron oxide nanoparticles will be used as the carrier of the photosensitizer because of: their ability to carry and deliver therapeutic photosensitizers into deep-seated tumours; the enhanced solubility of the hydrophobic PS with an appropriate size to accumulate in the tumour tissues *via* enhanced permeability and retention effect; and the ability to attack cancer cells selectively without harming other healthy cells. The Fe_3_O_4_ NPs will be functionalized with chitosan, which is a biodegradable, biocompatible polysaccharide and, in comparison with many other polymers, has many free –OH and –NH_2_ groups that can serve as anchors for conjugation of therapeutics and targeting ligands.

Considering the above mentioned information, we focused our research on attaching functionalized ZnPc with carboxylic groups (–COOH) to an Fe_3_O_4_/chitosan system hoping to get a synergistic effect in the photodynamic parameters of the resulting composite.

## Materials and methods

### Materials

All materials, trimellitic anhydride, zinc acetate dehydrate (Zn(CH_3_COOH)_2_·2H_2_O), ammonium molybdate tetrahydrate ((NH_4_)_6_MO_7_O_24_)·4H_2_O, anhydrous sodium sulfate (Na_2_SO_4_), urea, 1-bromo-naphthalene, hydrochloric acid and acetic acid were purchased from Sigma-Aldrich, Inc., St. Louis, MO, USA and used as received. Dimethyl sulfoxide (DMSO), chitosan (Ch) and hydrogen peroxide (H_2_O_2_) were obtained from Alfa Aesar, Heysham, UK. Fe_3_O_4_ nanoparticles (Merck) were cleaned in a flux of hydrogen at room temperature for 20 minutes.

### Equipment

The UV-vis spectra of the solutions were measured using a UV-vis spectrophotometer (Lambda 25, PerkinElmer, Inc., Shelton, CT, USA) from 200 nm to 1200 nm in 10 mm quartz cuvettes. The steady-state fluorescence spectroscopy was performed using a spectrometer (LS-55, PerkinElmer, Inc., Shelton, CT, USA) equipped with double-grating excitation and emission monochromators. Time-correlated single photon counting (TCSPC) was used to determine the fluorescence lifetime. The time-resolved fluorescence spectra were recorded on a spectrometer (FLS980, Edinburgh Instruments, Livingston EH54 7DQ, Oxford, UK). All the measurements were made at room temperature (295 ± 1 K). A Bruker D8 ADVANCE X-ray diffractometer (using Cu K_α_ radiation with *λ* = 1.5406 Å) was used for structural investigation of the magnetic nanoparticles. A Bruker FTIR spectrometer was used to provide information about the chemical composition.

## Synthesis

### The synthetic pathway of ZnPc(COOH)_4_

**Fig. 1:**
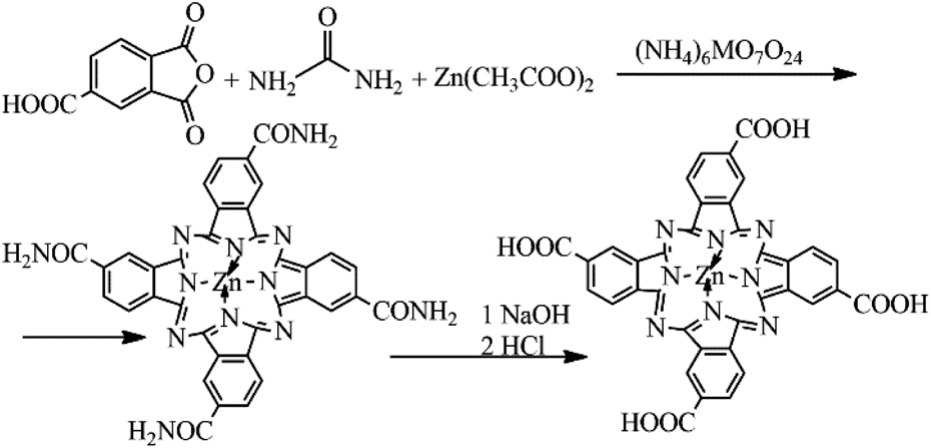
Schematic diagram of ZnPc(COOH)_4_ synthesis.

A mixture consisting of 4.35 g (0.022 mol) of trimellitic anhydride, 2.52 g of Zn(CH_3_COOH)_2_·2H_2_O, 0.3 g of ((NH_4_)_6_MO_7_O_24_)·4H_2_O, 0.5 g of Na_2_SO_4_, 13.51 g (0.225 mol) of urea and 5 ml of 1-bromonaphthalene was heated at 200–205 °C for 8 h with continuous stirring. After 8 hours, the reaction mixture was cooled to room temperature and treated with methanol. The obtained suspension was filtered. The solid reaction product was washed on the filter with methanol, chloroform and, finally, with acetone. After drying, the product was crumbled and then refluxed for one hour in 5% hydrochloric acid (HCl) solution. After drying, the same procedure was carried out with 5% sodium hydroxide (NaOH) solution for one hour at 90 °C. Finally, the solution was acidified with HCl until the pH was equal to 2, and the precipitated final product was filtered and dried in the open air. 0.68 g of ZnPc(COOH)_4_ was obtained with a yield of 70% (Fig. 1).

### Preparation of chitosan-functionalized magnetic nanoparticles

**Fig. 2:**
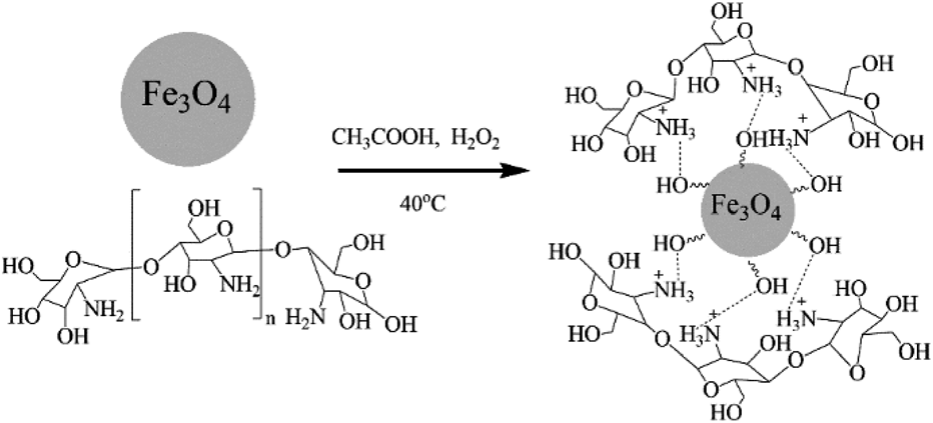
Schematic diagram of the preparation of chitosan-functionalized magnetic nanoparticles.

Chitosan and Fe_3_O_4_ were mixed in an appropriate proportion to form the chitosan–magnetic nanoparticles composite with amine groups by the reverse-phase suspension cross-linking method.^18^ Aqueous acetic acid solution was used as a solvent for the chitosan polymer and H_2_O_2_ was used as the cross-linker. In this specific procedure, a chitosan solution was prepared using a mixture of 2% acetic acid and 10% H_2_O_2_ solutions. Then 0.2 g Fe_3_O_4_ was added and stirred with strong ultrasonic agitation at room temperature for 4 h. At the end of this period, some of the chitosan-Fe_3_O_4_ nanocomposite particles were collected from the reaction mixture by using a permanent magnet. The product was washed with ethanol and dried in vacuum at 60 °C for 5 hours and used for XRD analysis (Fig. 2).

Chitosan is able to interact with negatively charged molecules,^19^ such as the hydroxyl (Fe–OH) groups on the surface of magnetite nanoparticles. The presence of –OH groups on the surface of the Fe_3_O_4_ nanoparticles was confirmed by the strong broad band with a maximum at 3431 cm^−1^ in the IR spectrum (Fig. 4), corresponding to *ν*(O–H) oscillations. We suppose that ionic interactions occur between the negatively charged CH_3_COO^−^ species and the positively charged (NH^3+^) groups of the chitosan molecules dissolved in the aqueous acetic acid solution.

### The conjugation of ZnPc(COOH)_4_ to chitosan, Fe_3_O_4_ and Fe_3_O_4_/Ch nanoparticles

Acetic acid is a weak acid and is a very common solvent for chitosan. A sample of 0.3 g of chitosan was dissolved in 50 ml of 2% concentrated acetic acid. Then 0.5 ml of 10% hydrogen peroxide was added to the solution for the destruction of intermacromolecular hydrogen bonds and interchain hydrogen bonds to make water-soluble chitosan. The appropriate ratio of chitosan to acetic acid in the chitosan–acetic acid solution was 1 : 0.5, and then ZnPc(COOH)_4_ was dissolved in a 1 : 1 DMSO/H_2_O solution. After that, both solutions were mixed, heated at 40 °C and stirred continuously for 40 min.

In a separate experiment, ZnPc(COOH)_4_ solution was mixed with a dispersion medium containing chitosan-functionalized magnetic nanoparticles at room temperature and stirred for 2 h using a mechanical stirrer.

Experiments where ZnPc(COOH)_4_ was dissolved in 1 : 1 DMSO/H_2_O solution and simply mixed with Fe_3_O_4_ were also performed.

## Results and discussion

### Structural analysis of the Fe_3_O_4_ and Fe_3_O_4_/chitosan magnetic nanoparticles

**Fig. 3:**
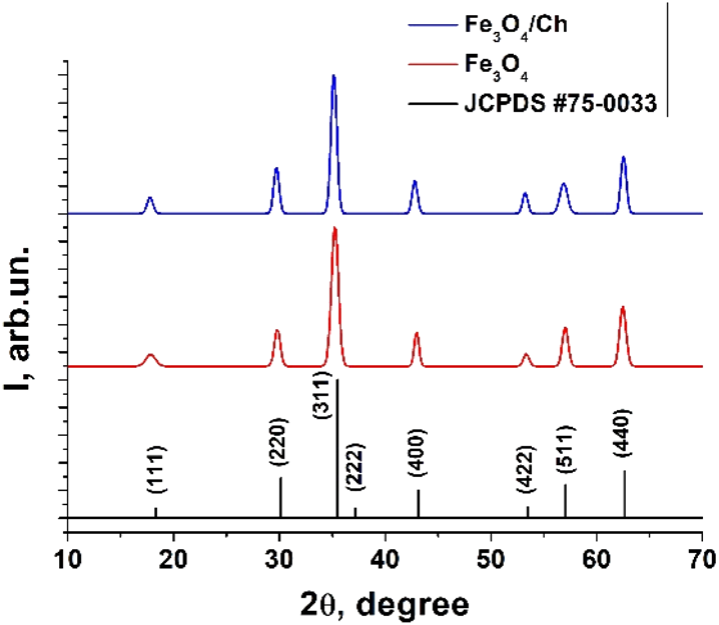
XRD patterns of the Fe_3_O_4_ and Fe_3_O_4_/chitosan nanoparticles, and the JCPDS #75-0033 card.

The X-ray diffraction patterns of the Fe_3_O_4_ and Fe_3_O_4_/chitosan nanoparticles, along with the standard pattern of Fe_3_O_4_ (JCPDS #75-0033), are shown in Fig. 3 and details of the peaks are given in Table 1. The similar XRD patterns reveal that Fe_3_O_4_ does not undergo any phase changes following functionalization with chitosan, a situation also confirmed by other reports.^20,21^

**Table 1:** Structural parameters of the Fe_3_O_4_ and Fe_3_O_4_/chitosan nanoparticles

	2*θ*	FWHM	*D*, nm	*D*_mean_, nm
Fe_3_O_4_	17.84	1.05	8.5	13.95
	29.81	0.67	13.7	
	35.25	0.79	11.	
	42.99	0.52	18.1	
	53.35	0.62	15.9	
	57.03	0.66	15.3	
	62.47	0.72	14.3	
Fe_3_O_4_/Ch	17.78	0.66	13.5	14.80
	29.72	0.61	15.0	
	35.16	0.66	14.1	
	42.81	0.60	15.8	
	53.24	0.59	16.7	
	56.88	0.86	11.7	
	62.54	0.61	16.8	

XRD diffraction analysis revealed a broad nature of the diffraction maxima, indicating that Fe_3_O_4_ has small crystallite sizes. The crystallite sizes were evaluated using the Debye–Scherrer formula:

1 *D* = (*kλ*/*β* cos *θ*)

where *λ* is the wavelength of the X-rays (1.5406 Å), *β* is the FWHM (full width at half maximum), *θ* is the diffraction angle, *k* = 0.94 and *D* is the crystallite size. The metal oxide nanoparticles have a mean crystallite size of 13.95 nm. During the coating process with chitosan, the crystallite size slightly increases, as the size of the individual crystallite is related to the thickness of the chitosan layer. The mean crystallite size of the nanoparticles with chitosan increases up to 14.80 nm.

### FTIR analysis

**Fig. 4:**
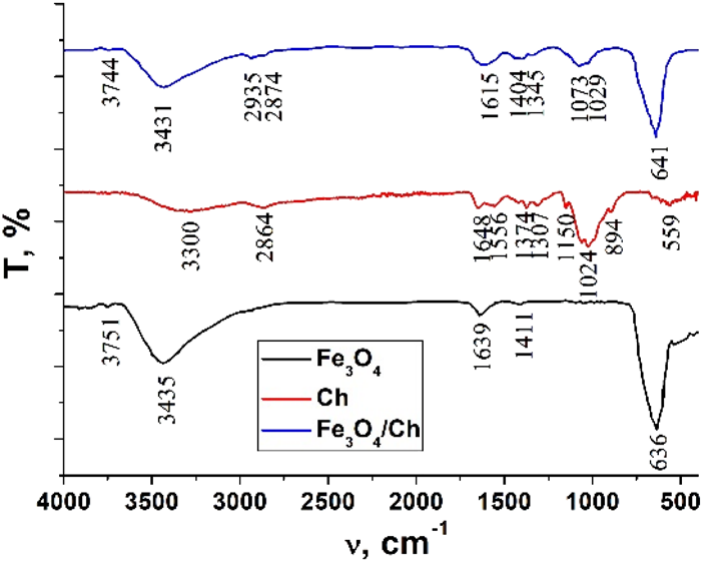
FTIR spectra of chitosan, the Fe_3_O_4_ nanoparticles and the Fe_3_O_4_/chitosan nanoparticles.

**Fig. 5:**
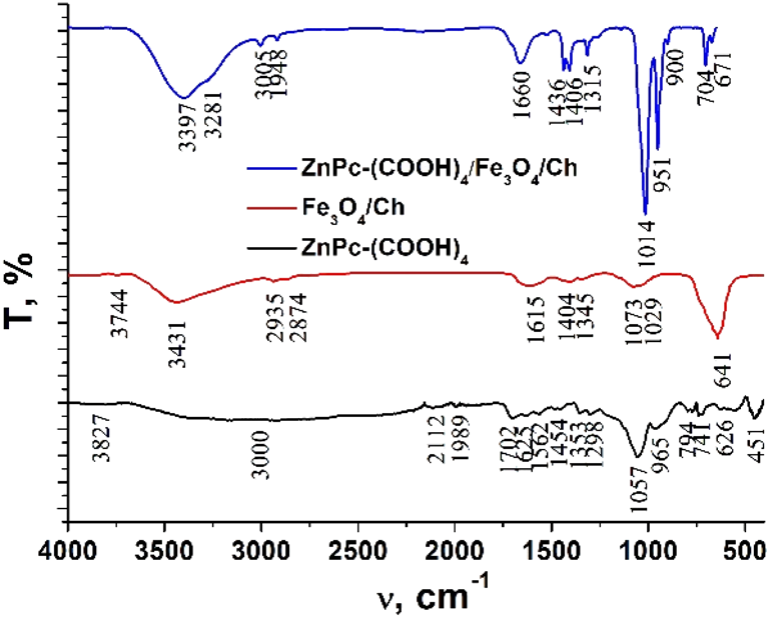
FTIR spectra of ZnPc(COOH)_4_, Fe_3_O_4_/chitosan and the ZnPc(COOH)_4_/Fe_3_O_4_/chitosan composite.

The FTIR spectra of chitosan, the Fe_3_O_4_ and Fe_3_O_4_/chitosan nanoparticles, ZnPc(COOH)_4_ and the ZnPc(COOH)_4_/Fe_3_O_4_/chitosan composite are presented in Fig. 4 and 5. The intense peak observed at 636 cm^−1^ in the FTIR spectrum of Fe_3_O_4_ is attributed to the stretching vibration mode associated with Fe–O bonds in the crystalline lattice of the Fe_3_O_4_ nanoparticles. This vibration is shifted to 641 cm^−1^ in the FTIR spectrum of the Fe_3_O_4_/chitosan nanoparticles. The modification and shift of the main characteristic bands (stretching C–O at 1024 cm^−1^, bending NH_2_ at 1648 cm^−1^, stretching O–H 3300 cm^−1^ and C–H 2864 cm^−1^) in the IR spectrum of chitosan to 1073 and 1029 cm^−1^ (*ν*(C–O)), 1615 cm^−1^ (*δ*(NH_2_)), 3431 cm^−1^ (*ν*(O–H)), and 2935 and 2874 cm^−1^ (*ν*(C–H)) in the IR spectrum of the Fe_3_O_4_/chitosan system) demonstrate the binding of chitosan to the Fe_3_O_4_ nanoparticles (Fig. 4). The result is consistent with similar investigations.^22,23^ The chemical interaction of ZnPc(COOH)_4_ with the Fe_3_O_4_/chitosan system is confirmed by the shift of the signal from 1702 cm^−1^ (*ν*(C=O)) of the protonated COOH groups in the IR spectrum of ZnPc(COOH)_4_, associated with splitting, to 1660 cm^−1^ (*ν*_sym_(COO)), and 1436 and 1406 cm^−1^ (*ν*_asym_(COOO)) that correspond to deprotonated carboxylic groups. This can be explained by the dissociation of carboxylic groups and formation of electrostatic interactions between NH_3_^+^ and COO^−^ fragments (Fig. 16a).

### UV-vis and fluorescence analysis

**Fig. 6:**
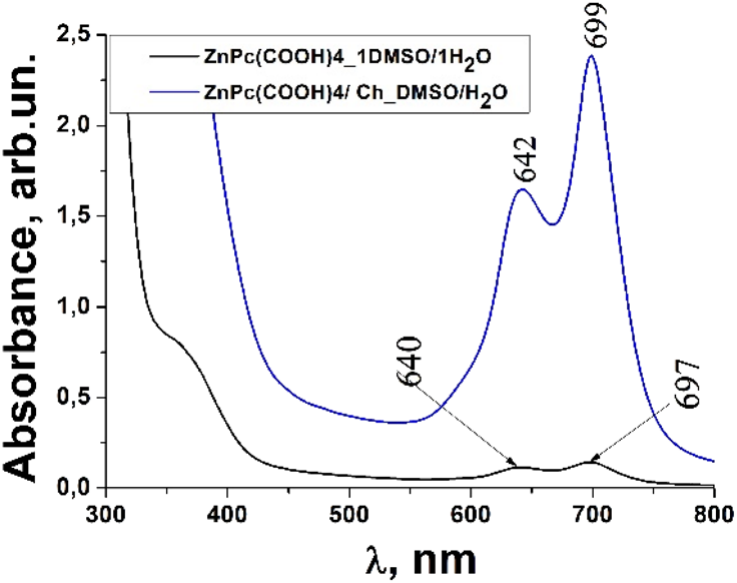
Absorbance spectra changes of the ZnPc(COOH)_4_ compound and ZnPc(COOH)_4_/Ch in DMSO/H_2_O solvent.

**Fig. 7:**
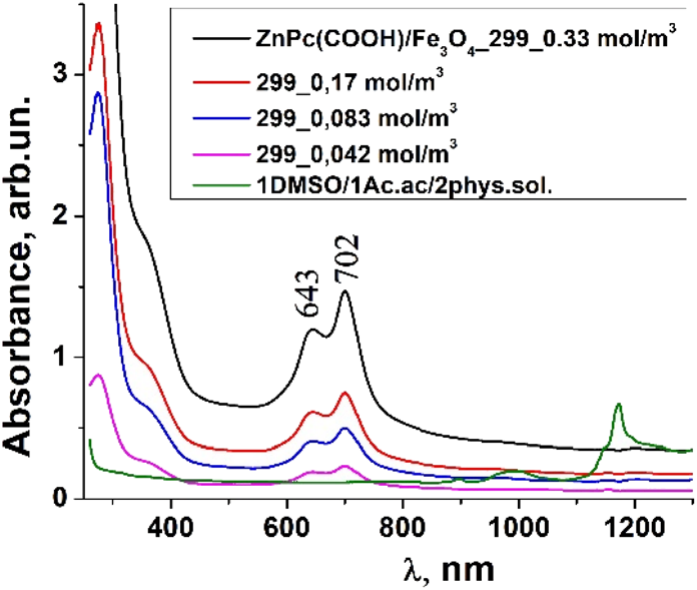
Absorbance spectra changes of ZnPc(COOH)_4_/Fe_3_O_4_ in DMSO/Ac.ac/phys.sol. solution, 1 : 1 : 2 ratio, at different molar concentrations.

**Fig. 8:**
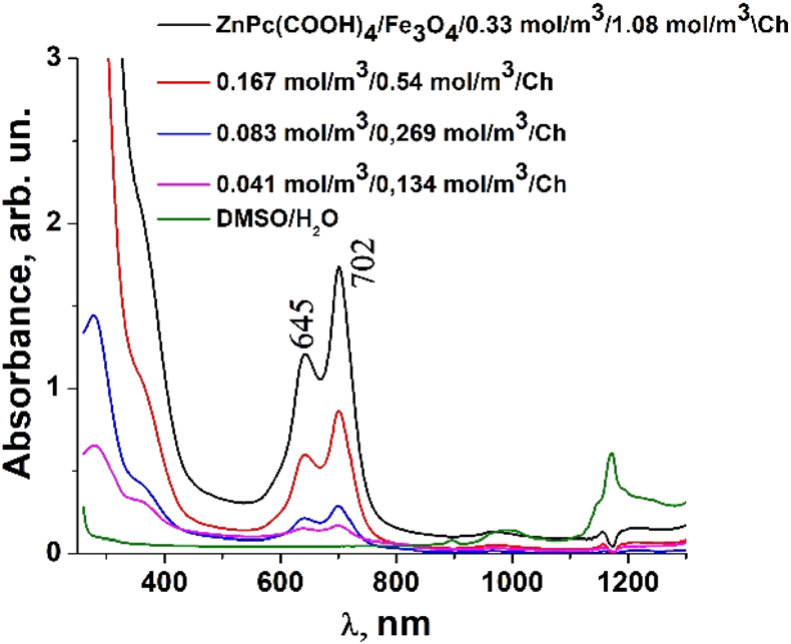
Absorbance of the ZnPc(COOH)_4_/Fe_3_O_4_/Ch composite in DMSO/H_2_O solution at different molar concentrations.

Usually, MPcs give rise to electronic spectra with two strong absorption bands, one around 300 nm, called the “B” or Soret band, due to electronic transitions from the deeper π-HOMO to n*-LUMO energy levels, while the other at 600–650 nm, called the “Q” band, due to electronic transitions from the π-HOMO to π*-LUMO energy levels.^24^ The UV-vis spectra of ZnPc(COOH)_4_ and ZnPc(COOH)_4_/Ch in DMSO/H_2_O are presented in Fig. 6. The absorption spectra of the synthesized materials display absorption peaks in the visible region at around 700 nm. In the case of ZnPc(COOH)_4_ and ZnPc(COOH)_4_/Ch dissolved in DMSO/H_2_O, absorption maxima are situated at 640 and 697 nm, and 642 and 699 nm, respectively. The shifts of the Q band depend on the change in electron distribution in the phthalocyanine ring caused by the substituents and their position. The UV-vis spectra of ZnPc(COOH)_4_ immobilized on non-functionalized magnetic nanoparticles are shown in Fig. 7. The intensity of the absorption for the ZnPc(COOH)_4_/Fe_3_O_4_ material in a solution of 1 : 1 : 2 DMSO/Ac.ac/phys.sol. (where “Ac.ac” is acetic acid, and “phys.sol.” is a physiological NaCl solution) decreases when decreasing the molar concentration of ZnPc(COOH)_4_ from 0.33 mol m^−3^ to 0.042 mol m^−3^. The conjugation of ZnPc(COOH)_4_ with the chitosan-functionalized Fe_3_O_4_ nanoparticles gives rise to similar positions of the maxima in the Q band absorbance spectra (Fig. 8) as in the case when ZnPc(COOH)_4_ is mixed with the non-functionalized Fe_3_O_4_ nanoparticles. A difference appears in the width of the bands, with a narrower band being found for ZnPc(COOH)_4_/Fe_3_O_4_/Ch than for ZnPc(COOH)_4_/Fe_3_O_4_.

**Fig. 9:**
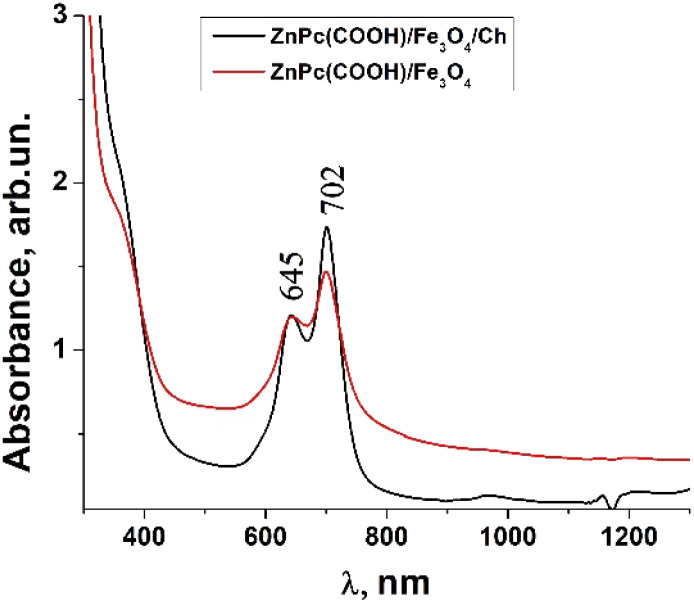
Comparison of the absorbance of the ZnPc(COOH)_4_/Fe_3_O_4_ and ZnPc(COOH)_4_/Fe_3_O_4_/Ch composites in DMSO/phys.sol. 0.9% and DMSO/H_2_O_2_/Ac.ac solutions.

In Fig. 7, Fe_3_O_4_ was dissolved in a physiological solution of 0.9% NaCl and 2% acetic acid. For the Fe_3_O_4_/chitosan solution measurements shown in Fig. 8, 2% acid acetic and 10% hydrogen peroxide were used. The Q band extends into the 580–800 nm region and exhibited two peaks at *λ*_max_ = 645 nm and 702 nm in the case of the Fe_3_O_4_ nanoparticles linked to chitosan (Fig. 8), almost the same values as when Fe_3_O_4_ is not bound to chitosan (Fig. 7). Both the ZnPc(COOH)_4_/Fe_3_O_4_/chitosan and ZnPc(COOH)_4_/Fe_3_O_4_ spectra (Fig. 9) show similar specific absorption peaks of the phthalocyanine aromatic ring. The chitosan had no obvious absorption peak in the visible region, but leads to an increased intensity of the 702 nm peak and a narrower Q band. The comparison in Fig. 9 allows us to suppose that the Q absorption band could be assigned to the π–π* transition on the ZnPc macrocycle. Introducing the peripheral –COOH substituent onto the macrocycle of ZnPc led to a significant bathochromic shift of the absorption spectra due to an increased destabilization of the HOMO electron state *versus* the LUMO state.

The low energy peak is due to the monomer, while the high energy peak is caused by the aggregation. The aggregation species persisted more when the Fe_3_O_4_ nanoparticles were not bound to chitosan.

**Fig. 10:**
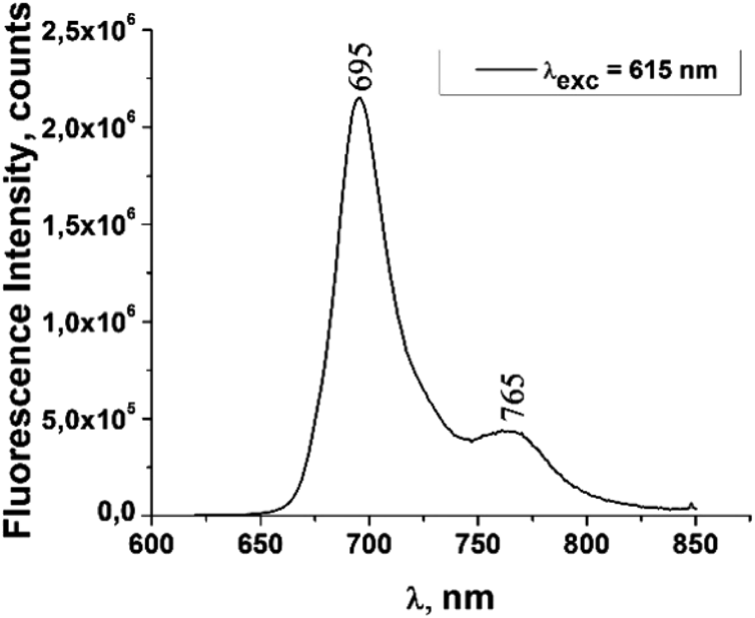
Emission spectrum of ZnPc(COOH)_4_ in DMSO/H_2_O solution.

**Fig. 11:**
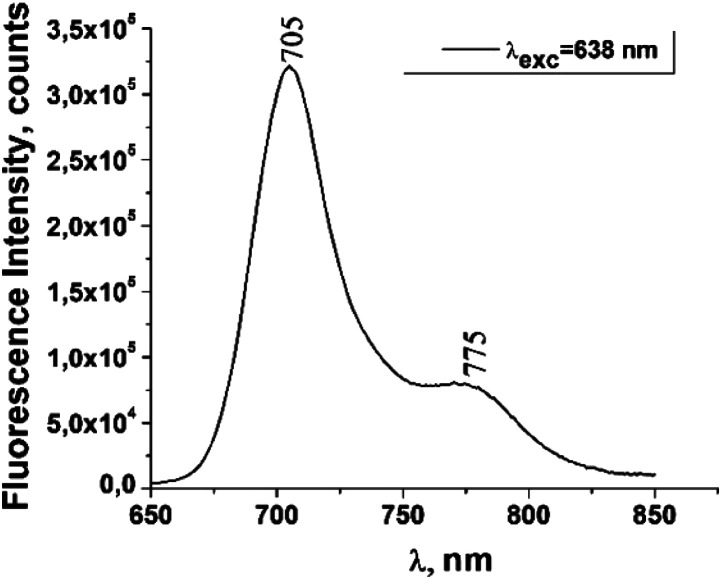
Emission spectrum of ZnPc(COOH)_4_/chitosan in DMSO/H_2_O solution.

**Fig. 12:**
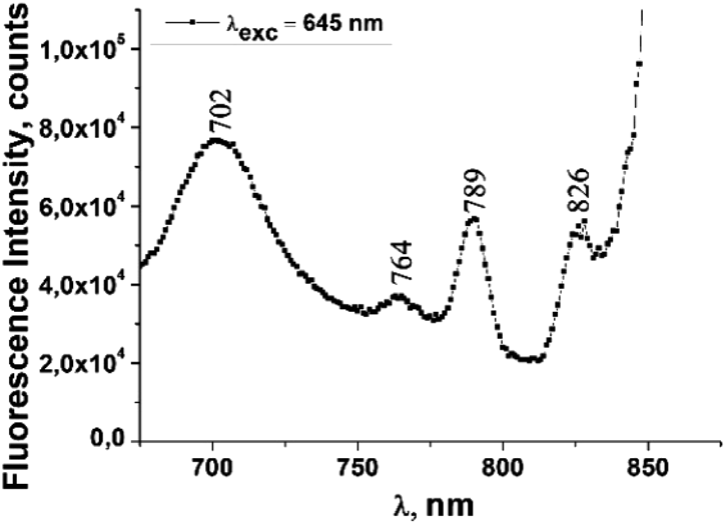
Emission spectrum of ZnPc(COOH)_4_/Fe_3_O_4_ in DMSO/phys.sol. 0.9% solution.

**Fig. 13:**
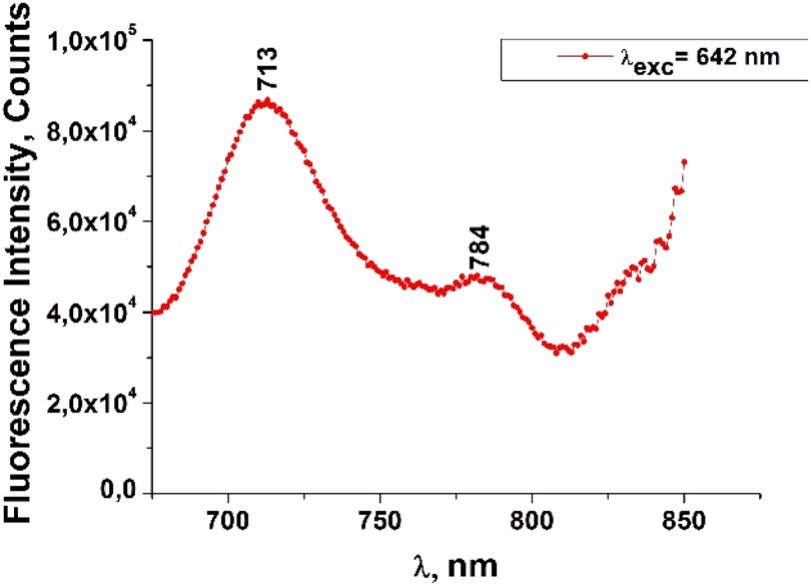
Emission spectrum of ZnPc(COOH)_4_/Fe_3_O_4_/chitosan in DMSO/H_2_O_2_/Ac.ac solution.

The fluorescence emission spectrum of ZnPc(COOH)_4_ in DMSO/H_2_O is shown in Fig. 10. The fluorescence spectrum after excitation at 615 nm shows two emission bands situated at 695 nm and 765 nm. The fluorescence spectrum of the ZnPc(COOH)_4_/chitosan system (Fig. 11) after excitation at 638 nm also shows two bands, as in Fig. 10, but they are both shifted 10 nm into the near-infrared region. The fluorescence spectrum of ZnPc(COOH)_4_ immobilized on the Fe_3_O_4_ magnetic nanoparticles shows broad and structured fluorescence at 702 nm, 764 nm, 789 nm and 826 nm, and shows an increase in intensity at 850 nm, when excited at 645 nm (Fig. 12). The limits of the measurement equipment did not allow us to record fluorescence above 850 nm. The spectrum of ZnPc(COOH)_4_ immobilized on the Fe_3_O_4_/chitosan magnetic nanoparticles shown in Fig. 13 displayed less structured fluorescence. Only two broad bands situated at 713 nm and 784 nm shifted to the near-infrared region are revealed. The resultant red-shift was associated with the electrostatic interaction between ZnPc(COOH)_4_ and the chitosan-functionalized Fe_3_O_4_ nanoparticles.

**Fig. 14:**
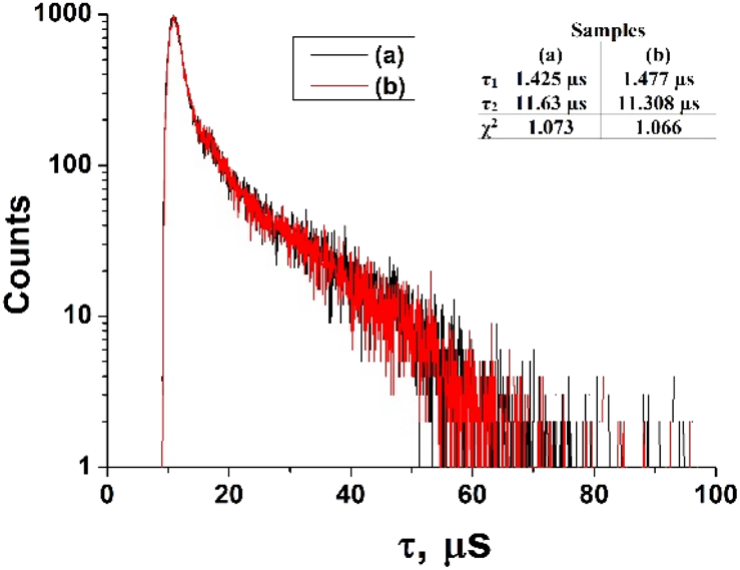
The fluorescence lifetimes of ZnPc(COOH)_4_ (a) and ZnPc(COOH)_4_/chitosan (b) in DMSO/H_2_O solution.

The fluorescence lifetimes of ZnPc(COOH)_4_ and ZnPc(COOH)_4_/chitosan in DMSO/H_2_O solution are presented in Fig. 14.

The fluorescence decays of ZnPc(COOH)_4_ and ZnPc(COOH)_4_/chitosan in DMSO/H_2_O solutions at about the same interval of excitation wavelengths (*λ*_exc_ = 684–772 nm) show a bi-exponential behaviour with lifetime values being 1.42 μs and 11.63 μs for the solution without chitosan, while, for the solution with chitosan, values of 1.47 μs and 11.31 μs are reported. The presence of chitosan does not induce significant variations in the fluorescence decay times. Thus, because the NH_2_ groups in chitosan can form the same ionic bonds with the COOH groups, even in the absence of Fe_3_O_4_, but only in the presence of Fe_3_O_4_, the fluorescence decay time changes. So, the presence of Fe_3_O_4_ NPs is vital for the main result of the chemical reactions of the developed composite.

**Fig. 15:**
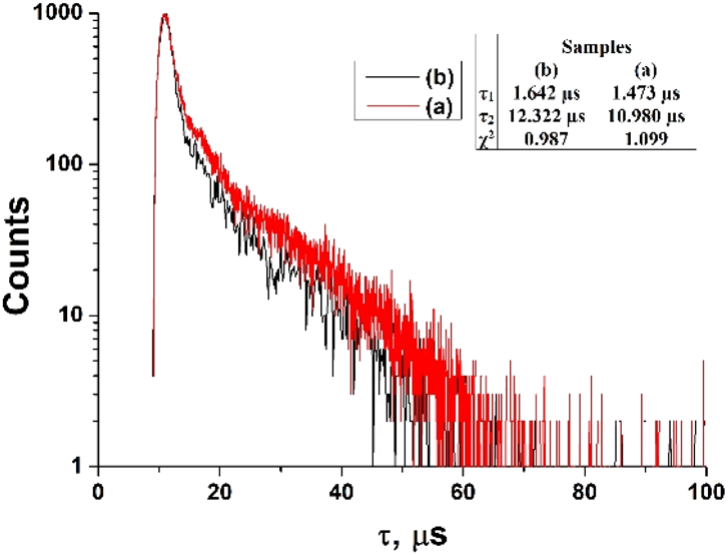
The fluorescence lifetimes of ZnPc(COOH)_4_/Fe_3_O_4_ in DMSO/phys.sol. 0.9% (a) and ZnPc(COOH)_4_/Fe_3_O_4_/chitosan in DMSO/H_2_O2/Ac.ac (b) solutions.

In Fig. 15, the fluorescence lifetimes of ZnPc(COOH)_4_/Fe_3_O_4_ and ZnPc(COOH)_4_/Fe_3_O_4_/chitosan in DMSO/phys.sol. 0.9% and DMSO/H_2_O_2_/Ac.ac solutions are illustrated. The fluorescence decay curve for ZnPc(COOH)_4_/Fe_3_O_4_ yielded two lifetimes of 1.47 μs and 10.98 μs. The values are smaller than those of the ZnPc(COOH)_4_/Fe_3_O_4_/chitosan composite, although the contribution of the shorter lifetime component is less than 13%. The longer lifetime in the ZnPc(COOH)_4_/Fe_3_O_4_/chitosan composite can be explained by the magnetic nanoparticles’ functionalization with chitosan. The fluorescence quantum yield values of the ZnPc(COOH)_4_/Fe_3_O_4_ or ZnPc(COOH)_4_/Fe_3_O_4_/chitosan composites in DMSO/phys.sol. 0.9% and DMSO/H_2_O_2_/Ac.ac solutions of 1 : 1 ratio were found to be lower than that for non-substituted ZnPc in non-diluted DMSO (0.67 in ref. 25) due to increased intersystem crossing in the presence of the Fe_3_O_4_ NPs. The photophysical parameters: triplet (*Φ*_T_) quantum yields and the triplet-state lifetimes (*τ*_T_), for ZnPc(COOH)_4_ linked to magnetic nanoparticles are presented in Table 2.

**Table 2:** Photophysical parameters: triplet quantum yields (*Φ*_T_) and the triplet-state lifetimes (*τ*_T_) for all compounds

	Solvent	*λ*_abs_, nm	*λ*_emis_, nm	*Φ*_T_	*τ*_T_, μs
ZnPc(COOH)_4_	DMSO/H_2_O	697	765	0.25	11.63
ZnPc(COOH)_4_/chitosan	DMSO/H_2_O	699	775	0.27	11.31
ZnPc(COOH)_4_/Fe_3_O_4_	DMSO/phys.sol. 0.9%	702	826	0.23	10.98
ZnPc(COOH)_4_/Fe_3_O_4_/chitosan	DMSO/H_2_O_2_/Ac.ac	702	784	0.56	12.32

**Fig. 16:**
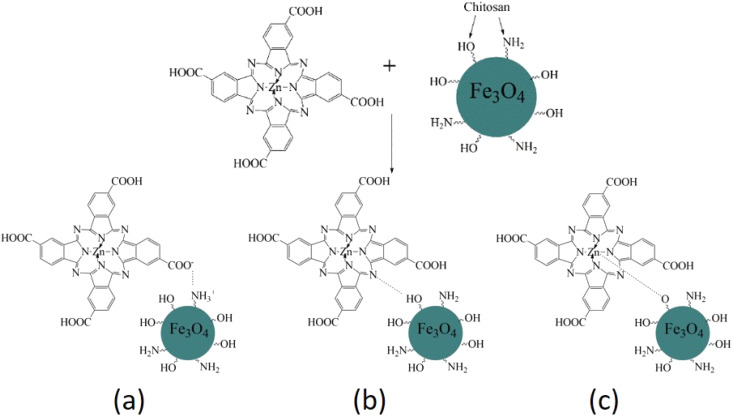
Scheme of the possible interactions (electrostatic (a), hydrogen (b) and coordination bonds (c)) of ZnPc(COOH)_4_ with the chitosan-functionalized Fe_3_O_4_ nanoparticles.

So, we suppose that the surface interaction between the amino groups of the chitosan/Fe_3_O_4_ and the carboxylic groups of ZnPc(COOH)_4_ most probably forms an electrostatic interaction. In addition to the electrostatic interaction between charged surfaces of ZnPc(COOH)_4_ and chitosan/Fe_3_O_4_, coordination bonds between the Zn^2+^ ions of phthalocyanine and the oxygen atoms of chitosan/Fe_3_O_4_ can be formed.^26,27^ Also, hydrogen bonds between the nitrogen atoms of phthalocyanine and the hydrogen atoms of chitosan/Fe_3_O_4_ are also possible, as shown in the scheme presented in Fig. 16.

So, significant efforts have been made to develop the ZnPc(COOH)_4_/Fe_3_O_4_/chitosan composite that has strong absorption of long-wavelength light and a triplet quantum yield of 0.56 that can be promising for PDT. But further studies will continue to improve the triplet-state lifetime and the triplet quantum yield, and elucidate the physiochemical processes in this composite. Moreover, *in vitro* and *in vivo* studies are required to elucidate the PDT effects.

## Conclusions

In this study we have developed:

(1) a soluble ZnPc(COOH)_4_ photosensitizer in DMSO/H_2_O, ratio 1 : 1, with high absorption at 702 nm, excitation/emission wavelengths of 615/765 nm and fluorescence lifetimes of 1.42 μs and 11.63 μs.

(2) Fe_3_O_4_/chitosan magnetic nanoparticles with a mean crystallite size of the nanoparticles up 14.80 nm using the suspension cross-linking technique.

(3) ZnPc(COOH)_4_ immobilized on chitosan-functionalized Fe_3_O_4_ nanoparticles through an immersion method with the aid of DMSO/H_2_O_2_/Ac.ac solution, exhibits higher triplet lifetimes of 1.6 μs and 12.3 μs.

The values of the triplet quantum yield (0.56) and the triplet-state lifetimes of ZnPc(COOH)_4_/Fe_3_O_4_/Ch make this composite a promising candidate for PDT.

## Author contributions

Conceptualization: T. Potlog, S. Robu and I. Bulimestru; methodology: A. Popusoi and S. Robu; formal analysis: A. Popusoi; investigation: I. Lungu and A. Popusoi; data curation: A. Popusoi and I. Lungu; writing – original draft preparation: T. Potlog and S. Robu; writing – review and editing: I. Bulimestru, T. Potlog and S. Robu; supervision: T. Potlog. All authors have read and agreed to the published version of the manuscript.

## Conflicts of interest

The authors declare no conflict of interest.

## Supplementary Material
